# Development and implementation of water safety plans for groundwater resources in the southernmost city of West Azerbaijan Province, Iran

**DOI:** 10.1007/s40201-020-00488-7

**Published:** 2020-06-08

**Authors:** Kazhal Masroor, Majid Kermani, Mitra Gholami, Farzad Fanaei, Hossein Arfaeinia, Sepideh Nemati, Masoumeh Tahmasbizadeh

**Affiliations:** 1 Department of Environmental Health Engineering, School of Public Health, Tehran University of Medical Sciences, Tehran, Iran; 2 Research Center of Environmental Health Technology, Iran University of Medical Sciences, Tehran, Iran; 3 Department of Environmental Health Engineering, School of Public Health, Iran University of Medical Sciences, Tehran, Iran; 4 Student Research Committee, School of Public Health, Iran University of Medical Sciences, Tehran, Iran; 5 Systems Environmental Health and Energy Research Center, The Persian Gulf Biomedical Sciences Research Institute, Bushehr University of Medical Sciences, Bushehr, Iran; 6 Department of Environmental Health Engineering, Faculty of Health and Nutrition, Bushehr University of Medical Sciences, Bushehr, Iran; 7 Department of Environmental Health Engineering, School of Public Health, Tabriz University of Medical Sciences, Tabriz, Iran; 8 Department of Environmental Health Engineering, School of Public Health, Isfahan University of Medical Sciences, Isfahan, Iran

**Keywords:** Drinking Water, Water safety plan, Assessment, Bukan City

## Abstract

The transfer of water from the source to the consumption point is always associated with the possibility of contamination in any of its various components. To resolve this problem, the World Health Organization has considered a water safety plan. The purpose of this study is to implement water safety plan in the water supply system of Bukan city. This study was performed on Bukan’s water supply system in 2019–20 using a software to guarantee the quality of the water safety plan and the WHO and IWA guidelines. The software checklists were prepared and after confirming the validity of the translation and its facial and content validity, it was completed based on the records of the Water and Sewerage Company and interviews with experts. Out of a total of 440 points of full-application of the program and 392 points for the reviewed phases, 183.6 points were acquired and 43.7% of WSP-coordinated implementation was observed. The highest percentage of WSP-coordinated implementation (75.2%) was assigned to the validation stage with the highest point, and the support program stage had the lowest percentage of performance (1.1%). Among the major components of the water supply system, the final consumption point received the most attention from the system. Given the lifespan of the introduction and use of WSP in the world, it was expected that better results would be obtained from evaluating the implementation and progress of this approach in Bukan’s water supply system. However, the implementation rate of this program in this city compared to other cities in Iran, showed that according to the implementation time (one year), the obtained results are relatively convincing and good and the water supply system has a moderate level of safety.

## Introduction

All living things on earth need water to survive [1]. Providing safe water today is one of the most important human challenges in societies, especially in developing countries [2, 3]. The largest populations in the world deprived of the blessings of having safe water are living in Asia and Africa and in the countryside. According to the WHO in 2017, approximately 2 billion people (3 out of 10) worldwide were denied access to safe and healthy water [4]. Delivery of safe water is not only dependent on achieving a high level of final quality of treated water; this is because water supply systems may meet this goal, but if there are hidden flaws in the design and operation, they can lead to various accidents [5]. Also, providing safe and high quality drinking water is the foundation of a healthy society and its economic development [6, 7]. Drinking water quality control in the diagnosis of disease agents and chemical pollutants using program monitoring and in accordance with national and international guidelines and standards, relies on the bacterial index and the maximum concentration of chemicals [8, 9]. These indicators are used as a means to convert complex information about water quality properties as a number that can be understood by officials and the public in order to determine the quality of consumed water according to the water quality indicators [10]. Since it is not possible to ensure water safety by simply testing water quality control tests, a systematic program can be used to ensure water safety [11]. Also, the water which is to be consumed as drinking water must be in accordance with the standards, which are provided by reputable national or global organizations [12]. The main purpose of qualitative studies of drinking water is to maintain public health and consumer health [13]. HACCP, QMRA and WSP are among the effective measures to protect the water supply system. The Water safety plan has been written and prepared by the World Health Organization and the International Water Institute in the third and fourth editions and it is a powerful tool to reduce risk and prevent water pollution from the catchment area to the point of consumption [14]. This program has been designed to manage health hazards that could threaten water resources. Preventive risk management requires that the risks associated with safe drinking water safety be identified, prioritized, and protected the drinking water quality before problems occur [15].

In 2020, Roberta Muoio et al. conducted a study on safety and risk assessment in Tuscany, Italy. In this study, a method for assessing water risk was proposed. Risk reduction was also assessed at each stage of the water treatment process train [16]. Van den et al. implemented a Water safety plan in 2019 in the Netherlands to evaluate water management methods [15]. Another study was conducted in 2017 by Setty et al. on the applications of the water safety plan in France and Spain. Evidence from this study shows that implementing a water safety plan can minimize the risks and threats affecting the water system [17]. In Iran, in the city of Qom, a study on the water safety plan was conducted by Shafiei et al. In 2017. In this study, by implementing the water safety plan, they were able to achieve a high level of safety of the water source system and distribution network [18]. In 2019, Razmju et al. conducted a study on risk assessment of Semnan city water supply system using water safety plan. According to this study, it was determined that the water supply system of semnan city has moderate safety [19].

According to the abovementioned issues, the purpose of this study is; 1) to determine the strengths and weaknesses of each step of the water safety plan in the quality management of drinking water and; 2) to determine the areas that need to be upgraded in Bukan water supply systems using the water safety plan guide and WSP QA TOOL software. This study is expected to reveal the need to change the current approach and highlight the role of WSP in improving the quality of drinking water by identifying the weaknesses and preventable points in water quality management in Bukan. It also contributes significantly to the risk-based decision-making structure of urban water supply systems, taking into account potential risks. The city is completely dependent on groundwater resources in terms of drinking water supply. Currently, the city’s water is supplied from underground sources with 4 wells outside the city and 8 deep wells inside the city with a capacity of about 600 Lit/s and enters the collection reservoirs with a storage capacity of 22,000 m^3^ through water transmission lines and, after chlorination, it will be provided to the citizens through the water distribution network with a length of about 309 km and 58,500 branches. 25% of the water extracted from these sources is not considered as water until it reaches the point of consumption, or water losses are removed from the consumption cycle.

## Materials and methods

### Study area

**Fig. 1:**
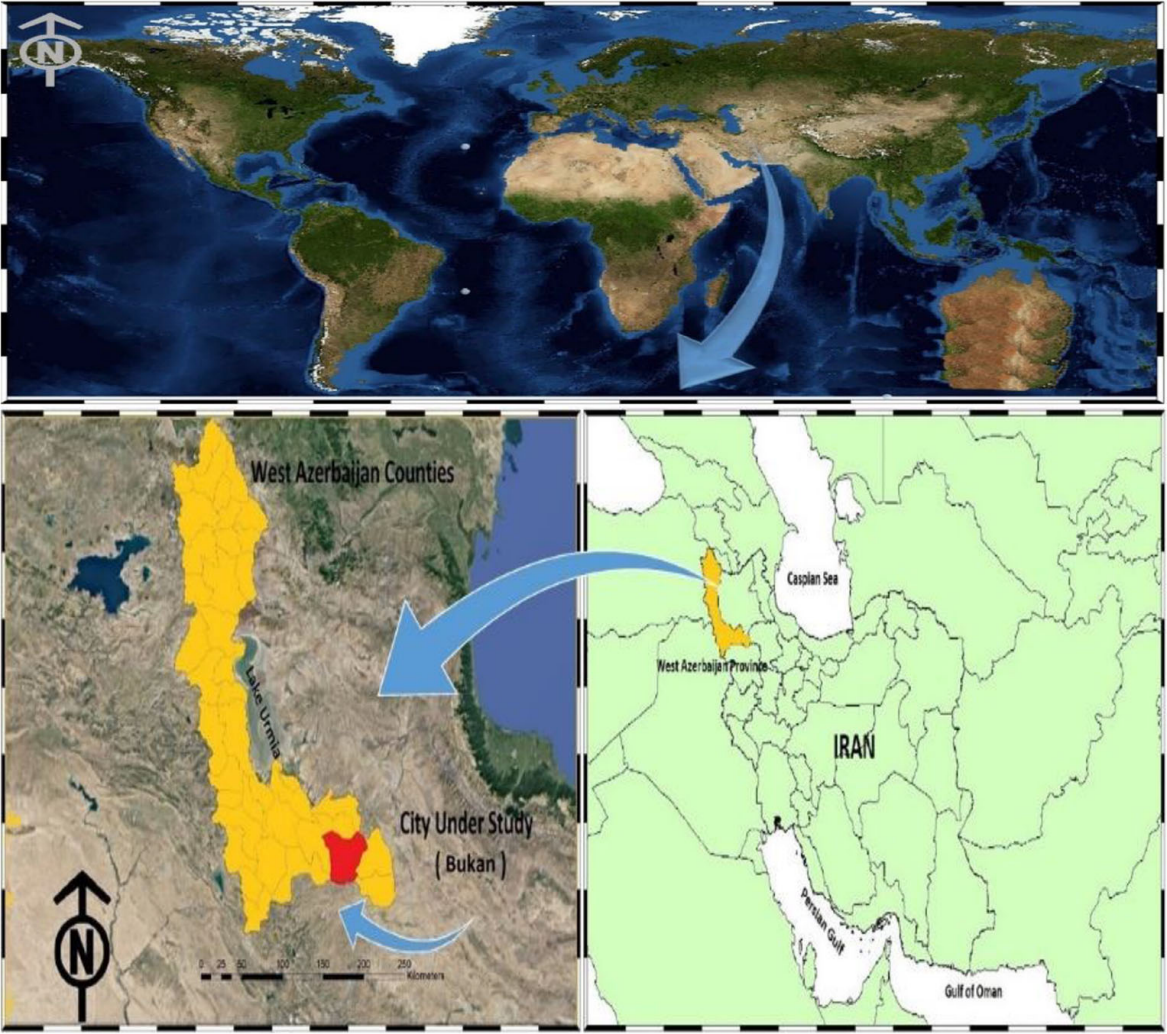
Geographical location of the study area

The study area is Bukan city, which is located in the south of West Azerbaijan province and south of Lake Urmia. (Fig. 1). The distance of this city from the center of the province is 185 km, 1370 m above sea level, the population is 251 thousand people and with an area of ​​2561 km^2^, which is about 6.5% of the province. This city is geographically located between 36 degrees and 31 min north longitude and 46 degrees and 12 min east longitude of the Greenwich meridian.

### Water safety plan

The Water safety plan offers a systematic approach to ensuring that the quality of water distributed to consumers is good and in line with standards. The inference of the water industry experience acquire is also through the development and implementation of water safety plans. To do this, it is necessary to pay attention to the water source, how to purify, store and distribute the treated water. The Water safety plan, based on a comprehensive risk assessment, includes the factors that can completely affect the quality of water distributed to consumers and addresses how to control and manage risk factors [20]. The Water safety plan has been written and provided by the World Health Organization in the third and fourth editions and, it is a powerful tool for preventative measures to ensure the quality of drinking water that uses risk assessment and risk management approach [21]. The Water Safety Plan is, in fact, a step-by-step guide to prevent dangerous water pollution management with the goal of providing safe drinking water that will attract the trust of consumers [22]. The primary purpose of the WSP is to describe, analyze the drinking water supply system (DWSS), identify all the factors that may cause a chemical, physical, microbial, and radiological hazard to water resources, reduce or eliminate these factors, and prevent water re-pollution during storage and distribution [23]. The WHO Framework for Drinking Water Safety, which is based on risk management and multi-barrier methods, offers many benefits to water suppliers, including: Helping to set priorities, regular structure for organizing risk management. Due to the program’s approach from the water source to the point of harvest, support, engagement and communication between the organization and various stakeholders is inevitable; making great efforts and justify decisions at all levels, from the lower levels to senior management; improving the understanding of the system, which reduces uncertainty in decisions, increases the credibility of the system [24].

### Data and collection

Initially, the WSP QA tool water quality safety software (developed by the World Health Organization) and its related checklists were prepared and, it was completed through an interview with one of the experts of the Water and Sewerage and Regional Water Affairs Organization of Bukan city. After completing the relevant checklist, the answers of each section entered the WSP QA Tool software and the related results were examined in the form of graphs and tables. Each of the water resources, transmission and distribution systems and consumption points includes eight parts: a) stakeholder identification b) Hazard identification c) risk assessment d) control measures and validation e) Improvement plan f) operational monitoring g) Management procedures and H) WSP revision. This software has been written and available in English, French, Japanese, Laos, Spanish and Vietnamese languages ​​and we will enter the main parts of the software by selecting the desired language. The next part of the software includes a general overview of the tools and general instructions on how to use the tools. In this section, general explanations about the software are provided and it includes the general view and general structure of the software and who, how and when should use this software. The next section is where the results of WSP-related assessment information are introduced. In this section, the input information has been divided into 2 parts, which include 12 tables. Each table contains a certain number of questions and options, and each question contains a guide on how to answer it. For this purpose, checklists consisting of 85 questions have been prepared separately for the phases and records and information registered in Bukan City Water and Sewerage Company, regional water affairs and interviews with the employees of this organization were completed to answer and record information in these checklists. The recorded information and the obtained data are introduced to WSP QA Tool software in both quantitative and qualitative forms. Thus, in the section related to entering data in the software environment, there are 12 tables appropriate to the implementation phases of the program, each of which has a certain number of questions. The answers to the relevant questions in the software are recorded quantitatively and in the same way as the obtained ones, but the necessary answers to the questions entered based on the scoring system according to the software work guide. In this system, scoring is based on the implementation of each stage, from zero to 4. The possible raw point, the point obtained in this study, and the implementation progress rate in terms of percentage for each phase are determined based on the specified relationships in the software.

Tools can be adapted to the specific needs of individuals. In particular, new questions can be added to the tool using the “Create a New Question” button. Two types of questions can be added: one is a general question which is not pointd and the other is the assessment question whose point is added to the total point of the table to which the question has been added. To distinguish between these new questions and the current tool questions, the text of the new questions with a different color font is recorded in the list of questions. It is important to consider all the questions, even if no specific activity has been started yet. In addition, in line with the WSP guidebook approach, the questions are consecutive, and must be completed before each subsequent step is completed. In the assessment results section, the results are presented in the form of graphs and tables after analyzing the input data. These diagrams and summary tables will help the water supplier understand their overall performance. After completing the assessment process, by clicking on the “View Results” button, tables and output charts will appear. These summarized tables and charts will help the water supplier to easily identify where efforts should be targeted, where more resources are needed, and areas where progress has been made.

## Results

In this study, analysis was performed by WSP QA tool software in Excel environment after the introduction of the data and information extracted from the checklists. The implementation rate and progress of the water safety plan in the water supply system of Bukan city were shown in the form of tables and diagrams. Table 1 shows the steps of implementing and developing a complete WSP step by step, which evaluates the application and progress of the implementation of each step by answering the questions of the relevant checklists in accordance with the water safety plan. Then, the percentage of water safety plan-coordinated implementation for each stage is calculated as the point obtained (percentage implemented). In this study, the major components of the water supply system, namely the source, distribution network and endpoint, were also analyzed in software. Due to the lack of a water treatment plant in the city of Bukan, this part of the water supply system has not been evaluated and examined.

**Table 1:** Results of the general evaluation of the phases of the water safety plan by using software WSP QA TOOL for the Bukan city drinking water supply system

Steps to implement WSP	Number of questions	total raw score possible	Achieved score (Percentage executed)
WSP team	5	20	12.3–65%
System description	2	8	6.4–73%
Hazard identification and risk assessment	7	100	51–51%
Control measures and validation	5	68	31.6–48.2%
Improvement plan	3	48	–
Operational monitoring	4	64	25.42–42.7%
Verification	8	32	23.8–75.2%
Management procedures	3	36	19.22–52.9%
Supporting programmers	2	8	1.1–12%
Review of WSP	5	56	12.71–17%
TOTAL	44	440	183.6–43.7%

**Fig. 2:**
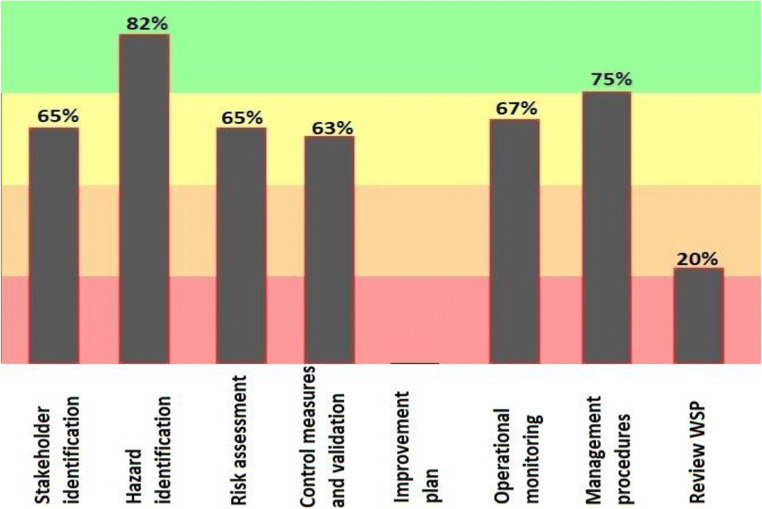
Results of the progress rate information of General implementation of water safety plan in main components source of drinking water supply in the city of Bukan (2019–2020)

In Fig. 2, the processes related to Hazard identification in the drinking water source obtained the highest point (82%) and the phase related to operation monitoring as well as processes related to management procedures, respectively with 67% and 75% of implementation coordinated with the water safety plan in the drinking water source of Bukan.

**Fig. 3:**
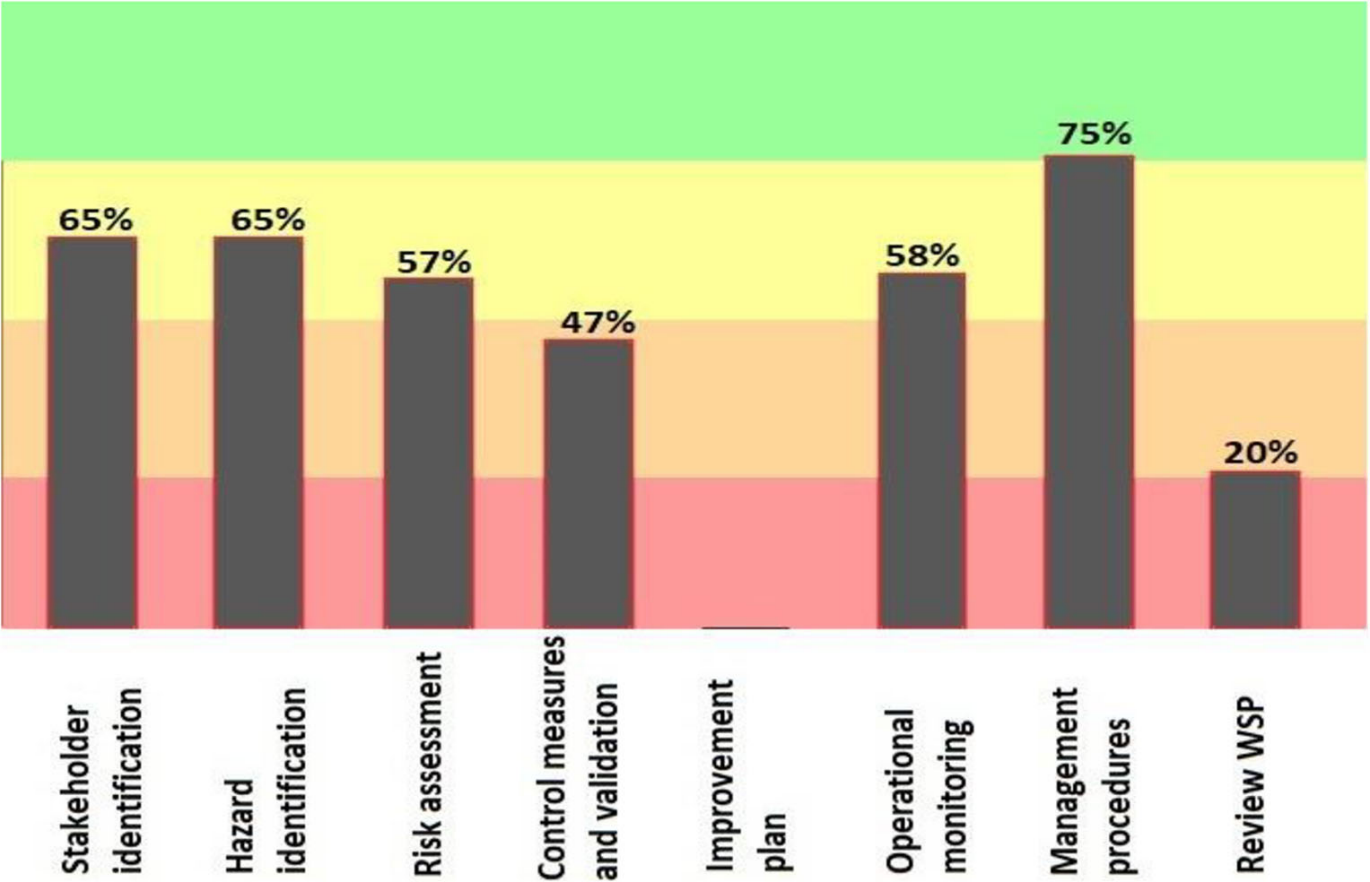
Results of the progress rate information of General implementation of water safety plan in main components distribution system of drinking water supply in the city of Bukan (2019–2020)

As shown in Fig. 3, in the Bukan Drinking Water Distribution Network, as in the case of the water source, the process of management procedures has 75% water safety plan-coordinated implementation also, scoring the stages such as stakeholders identifying and Hazard identification 65% matching with the program.

**Fig. 4:**
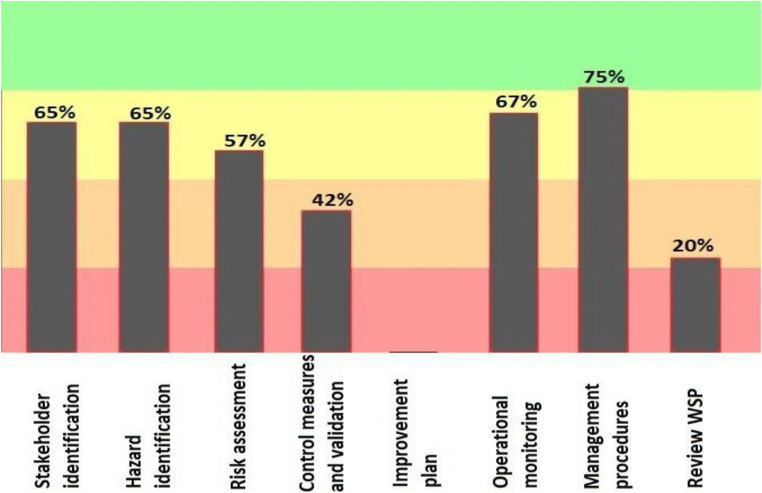
Results of the progress rate information of General implementation of water safety plan in main components Consumption point of drinking water supply in the city of Bukan (2019–2020)

Figure 4 shows that at the point of water consumption, the phase related to control measures and validation and review of the water safety plan relative to other evaluated parameters show a lower percentage (42%, 20% rrespectively) of accordance with the water safety plan.

**Fig. 5:**
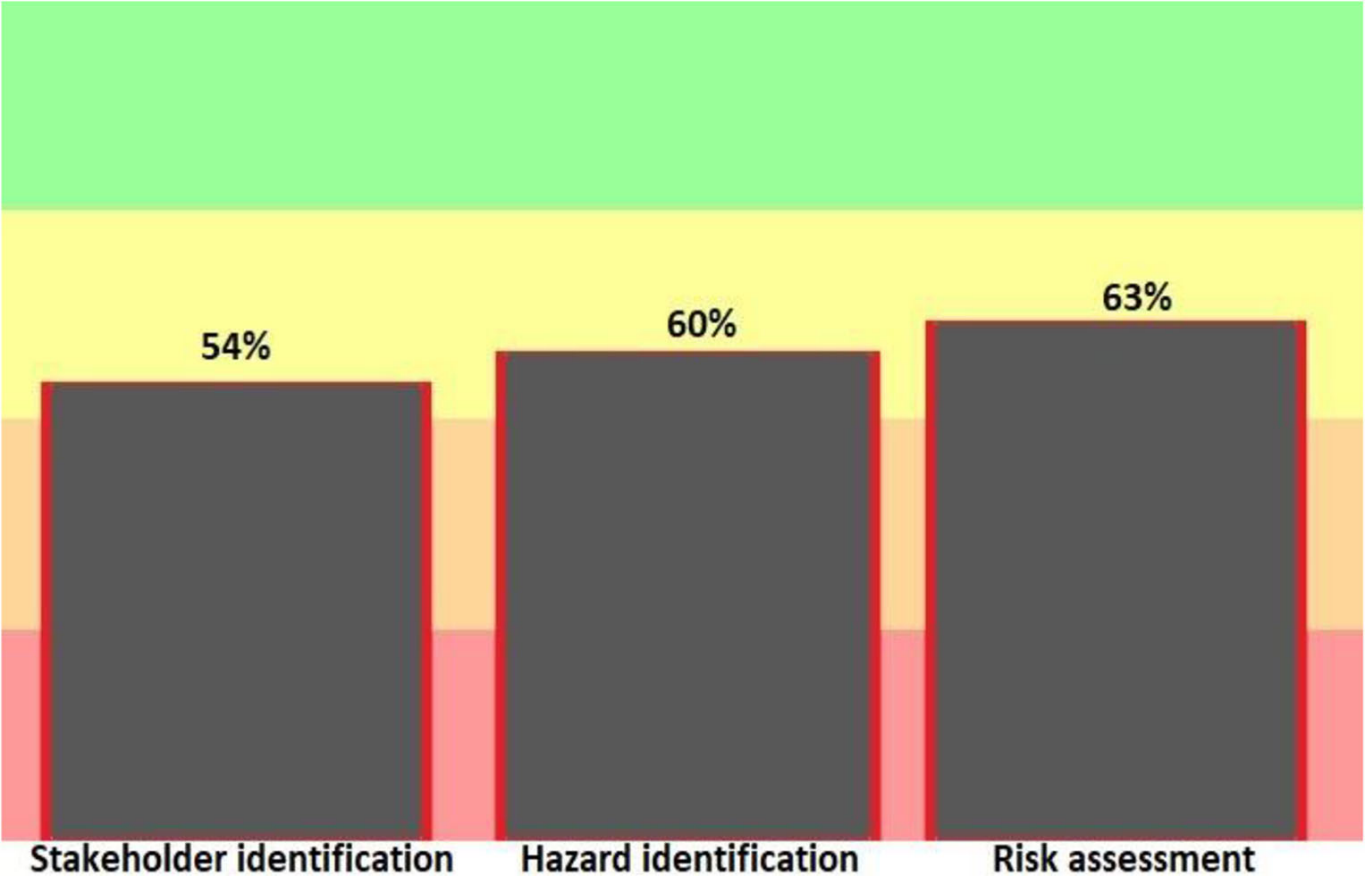
Results of the progress rate information of General implementation of water safety plan in hazard identification and risk assessment of drinking water supply in the city of Bukan (2019–2020)

Figure 5 shows the risk assessment phase and Hazard identification in water Bukan city. This step is done with three parameters: stakeholder identification and Hazard identification and risk assessment. According to this figure, this stage has obtained approximately 51 points out of 100 raw points and represents 58% of the executive progress rate in line with the WSP.

**Fig. 6:**
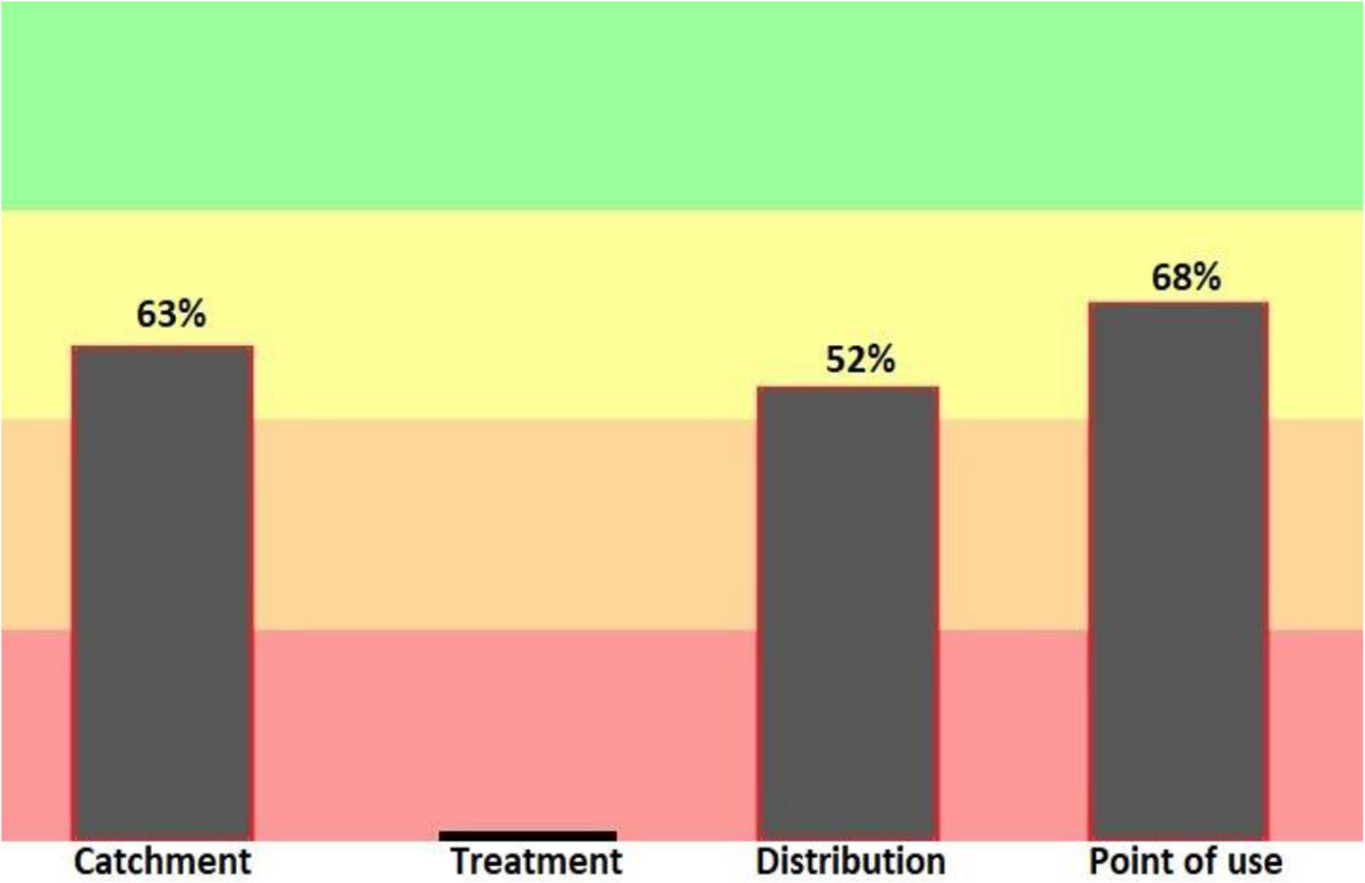
The progress rate of General implementation of water safety plan in main components of Drinking water supply system in the city of Bukan (2019–2020)

Figure 6 shows the output results related to the progress of each WSP process in terms of the major components of the Bukan city water supply system. Among the major components of the water supply system, water consumption is the most considered by the water supply system.

**Fig. 7:**
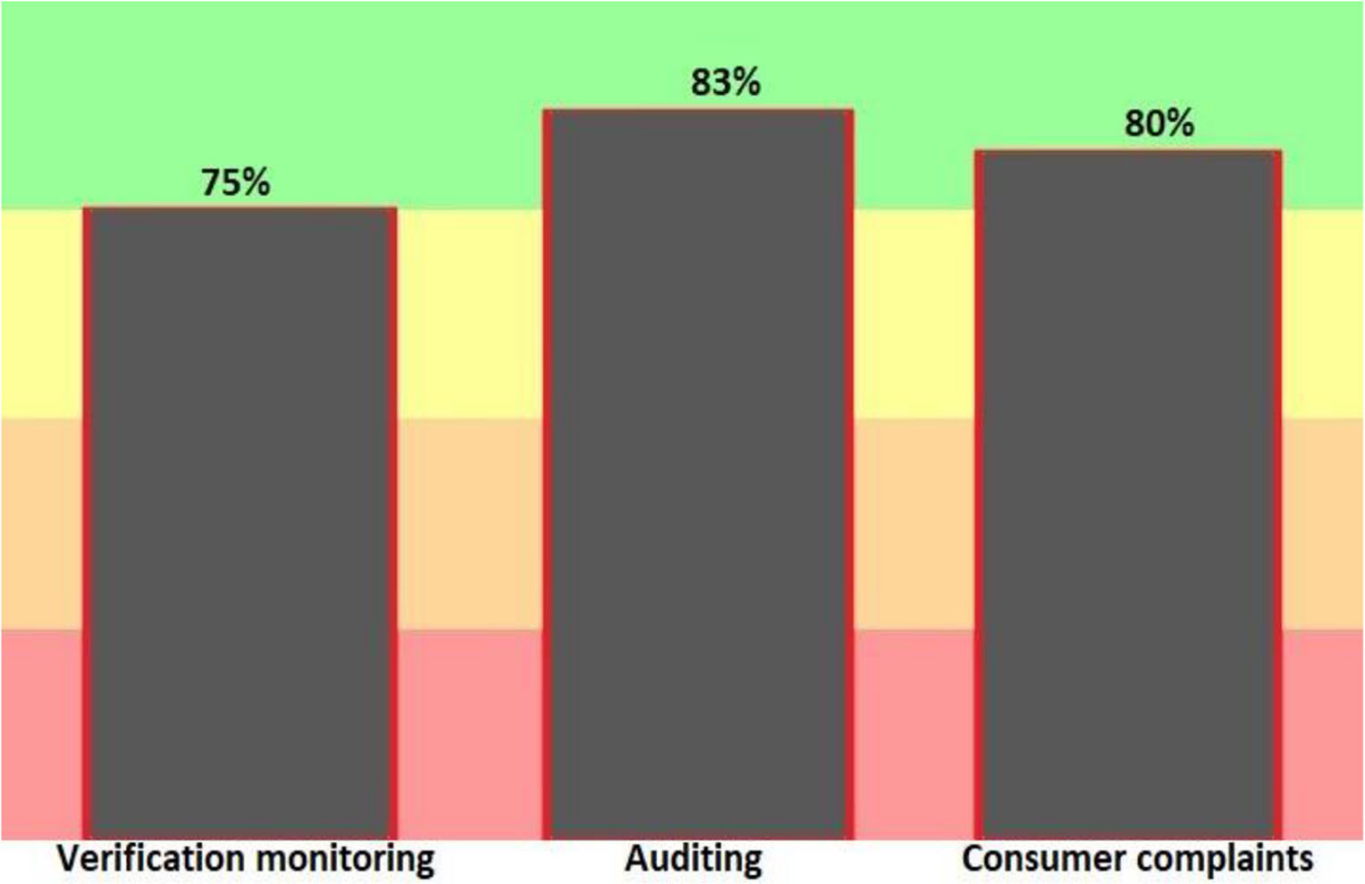
Results of the progress rate information of General implementation of water safety plan in Validation stage of drinking water supply in the city of Bukan (2019–2020)

One of the key parts of a water safety plan is to have an official process for validating and auditing WSP; because it ensures that the program works properly. Validation includes three activities of acceptability monitoring, internal and external audits of exploitation activities and consumer satisfaction. So these steps must be done simultaneously to provide evidence of effective WSP performance. According to Fig. 7 and Table 1, this step shows a 23.8% point, 75.2% performance alignment with the water safety plan.

## Discussion

Access to safe drinking water is a basic need and human right. The best way to ensure the quality of drinking water is to implement a comprehensive and integrated management system with the cooperation of all relevant organizations [22]. The new approach of the World Health Organization to achieve this goal is to implement a water safety plan. The purpose of the drinking water safety plan is to ensure the quality of drinking water based on risk management, which includes prevention of contamination of drinking water sources, treatment of water to reduce or eliminate pollution to meet standards, prevention of re-contamination of water during storage, distribution and consumption [25].

The results of Table 1 show that out of 440 total points of full program application and 392 points related to the studied phases, 183 points were obtained and 43.7% of the implementation was observed along with the WSP. Performance validation stage has had 23.8 points out of 32 possible points with the highest percentage of performance in line with the WSP program (78.13%), and the phase related to the support program with the lowest point (1.1 points out of 8 points) has had the lowest percentage of performance (12%). In the Bettina Rickert’s study, the highest risk assessment stage with the highest percentage of water safety plan-coordinated implementation was achieved [26]. On the other hand, in the study conducted by Setty et al. in southwestern France, the two stages of risk identification and control and validation criteria obtained the most points and get the most performance coordinated with the water safety plan [27]. Also in a study conducted by Gholami et al. on the safety assessment of drinking water supply in Khoy city; the validation phase accounted for the highest percentage of coordinated implementation (87%) and the support program phase achieved the lowest point (8.8%) [28]. In the study conducted by Aghaei and et al., leadership monitoring and control criteria had the most coordinated implementation [29]. Also, according to Gunnarsdottir, the highest implementation phase coordinated with the water safety plan was in the hazard identification network distribution network [22]. Also, a study by Hoshyari et al. on the water supply system of Hamadan city showed that performance, control measures and validation respectively with 78.13% and 48.53%, had the highest and lowest percentage of water safety plan-coordinated implementation [30]. Since the software is capable of evaluating some of the steps of the water safety plan, including the improvement / promotion program, it requires the full implementation of the program in the management of the water supply system and on the other hand, considering that in the city of Bukan, WSP is not fully implemented therefore, questions and points were related to phases that could not be evaluated; in the final analysis, it is not considered that this issue can be justified due to the software feature in the independent analysis of the data of each phase.

The table of system components shows the progress of the water safety plan in each component of the water supply system. In this sector, the water consumption point, with 68% of the total points, has received the most attention. According to a study conducted in Spain and France, the refinery and distribution network in France and the source of water supply in Spain had the highest point [17]. Settyv’s study in France identified risk identification as a key component of the water safety plan and in this regard, they emphasized that in order to achieve safe water, more attention should be paid to management at the harvest point [27].

According to the results of the software, the hazard identification section in the source was able to obtain 82% of the points. Risk assessment in the distribution network and the point of consumption gained equal and 65% points. According to the 2013 Progress Report on the Water safety plan, one of the goals of the program is to increase the safety of drinking water and identify and assess the hazards and hazardous events in the water supply system [31]. Figure 4 provides a more detailed overview of the three sections of stakeholder identification, hazard identification, and risk assessment. In this section, risk identification has gained the highest point (63%) and stakeholder identification has the lowest point (54%). In their 2017 study, Baum et al. noted the importance of risk assessment and said that effective risk management could help ensure safe drinking water and public health [32].

Effective WSP implementation helps maintain public health, improve leadership efficiency, and purposeful investment. For this purpose, it is necessary to develop mechanisms. According to it, the water supplier can realistically evaluate the implementation of WSP and identify the points of progress and areas in need of improvement [33]. Vietnam enumerated the benefits of implementing the Water Safety Plan for 12 years, including improving water quality, increasing consumer satisfaction, reducing waterborne diseases, and has ensured continued water supply [34]. According to the results of the overall assessment of WSP phases in Bukan, the weaknesses and vulnerabilities of the system are obvious, so that the main focus is on management procedures and Hazard identification, which in this regard is closely in agreement with the study conducted by Byleveld and Banda [35, 36]. On the other hand, the results of Mohammed Mustapha study in Nigeria, and Tavares were consistent with our study [37].

## Conclusion

The results of this study not only distinguish the areas and opportunities needed for promotion in the Bukan water supply system, but also specifies the inefficiency of the traditional approach. Given the overall application rate of the various WSP phases, as well as the attention of the Water Supply Organization to some key parameters such as risk identification and risk assessment in all three main parts of the water supply system, the system currently has a moderate level of safety. However, the potential for various pollutants in the water supply system can be minimized by focusing more on the steps of determining and validating control criteria, describing the system and leadership monitoring and increased the system’s flexibility to change the current quality management approach to the water safety plan.

## Abbreviations

WHO: World Health Organization
HACCP: Hazard Analysis Critical Control Point
QMRS: Quantitative Microbiological Risk Assessment
WSP: Water Safety Plan
DWSS: Drinking Water Supply System
